# Evaluating renal iron overload in diabetes mellitus by blood oxygen level-dependent magnetic resonance imaging: a longitudinal experimental study

**DOI:** 10.1186/s12880-022-00939-7

**Published:** 2022-11-18

**Authors:** Weiwei Geng, Liang Pan, Liwen Shen, Yuanyuan Sha, Jun Sun, Shengnan Yu, Jianguo Qiu, Wei Xing

**Affiliations:** Department of Radiology, Third Affiliated Hospital of Soochow University, 185 Juqian Street, Changzhou, 213003 Jiangsu China

**Keywords:** Diabetes mellitus, Diabetic nephropathy, Iron overload, Magnetic resonance imaging

## Abstract

**Background:**

Iron overload plays a critical role in the pathogenesis of diabetic nephropathy. Non-invasive evaluation of renal iron overload in diabetes in the management and intervention of diabetic nephropathy is of great significance. This study aimed to explore the feasibility of blood oxygen level-dependent (BOLD) magnetic resonance imaging (MRI) in evaluating renal iron overload in diabetes using a rabbit model.

**Methods:**

The rabbits were randomly divided into control, iron-overload (I), diabetes (D), and diabetes with iron-overload (DI) groups (each n = 19). The diabetes models were generated by injecting intravenous alloxan solution, and the iron-overload models were generated by injecting intramuscular iron-dextran. BOLD MRI was performed immediately (week 0) and at week 4, 8, and 12 following modeling. The differences in renal cortex (CR_2_^*^) and outer medulla R_2_^*^ (MR_2_^*^) and the ratio of MR_2_^*^–CR_2_^*^ (MCR) across the different time points were compared.

**Results:**

Iron was first deposited in glomeruli in the I group and in proximal tubular cells in renal cortex in the D group. In the DI group, there was iron deposition in both glomeruli and proximal tubular cells at week 4, and the accumulation increased subsequently. The degree of kidney injury and iron overload was more severe in the DI group than those in the I and D groups at week 12. At week 8 and 12, the CR_2_^*^ and MR_2_^*^ in the DI group were higher than those in the I and D groups (all *P* < 0.05). The MCR in the I, D, and DI groups decreased from week 0 to 4 (all* P* < 0.001), and that in the I group increased from week 8 to 12 (*P* = 0.034). CR_2_^*^ and MR_2_^*^ values displayed different trends from week 0–12. Dynamic MCR curves in the D and DI groups were different from that in the I group.

**Conclusion:**

It presents interactions between diabetes and iron overload in kidney injury, and BOLD MRI can be used to evaluate renal iron overload in diabetes.

## Background

Iron is an essential trace element. As a cofactor of several enzymes and the primary component of oxygen transporters, iron exerts important metabolic functions. Iron metabolism is closely related to numerous systemic diseases [1]. High dose iron intake or high iron storage can lead to oxidative stress and inflammatory reaction by increasing reactive oxygen species, which may lead to hyperglycemia, insulin resistance, and pancreatic β-cell dysfunction, and eventually leads to diabetes mellitus [2, 3]. Diabetes and iron overload possibly interact during the progression of diabetic nephropathy. Howard et al. [4] mentioned that urinary iron excretion increases in patients with diabetes. Dominguez et al. [3] reported on increased renal iron content in diabetic rats induced by streptozotocin. Moreover, Gao et al. [5] indicated that the combined effect of exogenous iron overload and diabetes on oxidative stress is a possible mechanism for aggravating kidney injury. Non-invasive evaluation of renal iron overload in diabetes in the management and intervention of diabetic nephropathy is of great significance.

T_2_^*^ is a magnetic relaxation property of any tissue, and is highly sensitive to iron loading [6]. T_2_^*^ technique is currently recognized as the preferred method for the non-invasive assessment of tissue iron deposition [7]. Blood oxygen level-dependent magnetic resonance imaging (BOLD MRI) is a T_2_^*^-based technique, and paramagnetic substances, such as iron and hemoglobin increase the transverse relaxation rate. This in turn increases the R_2_^*^ value (R_2_^*^ = 1/ T_2_^*^). Matsuo et al. [8] demonstrated that R_2_^*^ values were highly correlated with liver iron content in a murine model of iron overload. Moreover, R_2_^*^ values of BOLD MRI were related to the level of renal oxygenation, and indirectly reflected the degree of kidney fibrosis and injury [9, 10]. We hypothesized that BOLD MRI can be not only used to assess kidney iron deposition but also to indirectly evaluate kidney injury caused by renal iron overload in diabetic mellitus.

We aimed to explore the effect of diabetes and iron overload on kidney, and the feasibility of BOLD MRI in evaluating renal iron overload in diabetes.

## Materials and methods

This study was approved by the Ethics Committee for Animal Experimentation in the Third Affiliated Hospital of Soochow University (No. 2019-026) and was performed in accordance with the guideline and regulations for the Care and Use of Laboratory Animals and the ARRIVE (Animal Research: Reporting of In Vivo Experiments) guidelines.

### Animal model and animal subgroups

Seventy-six healthy New Zealand rabbits weighing 2–2.5 kg were randomly divided into four groups, namely the control (C), iron-overload (I), diabetes (D) and diabetes with iron-overload (DI) (n = 19 each) groups.

In the D and DI groups, alloxan monohydrate (ALX, Sigma-Aldrich Chemical, St Louis, Mo) was dissolved in 0.9% normal saline to achieve a concentration of 5%, and ALX solution (100 mg/kg) was administered intravenously via the marginal ear vein. 10% glucose water was given to the rabbits within 48 h after the ALX injection to help them get through the hypoglycemia period. 72 h after the injection of ALX solution, the blood glucose of peripheral blood of ear vein was detected by One Touch UltraEasy blood glucometer. Blood glucose levels ≥ 14.0 mmol/L supposedly reflect successful modeling of diabetes [11]. Rabbits in the C and I groups were injected with similar dose of 0.9% normal saline via the ear vein.

**Fig. 1:**
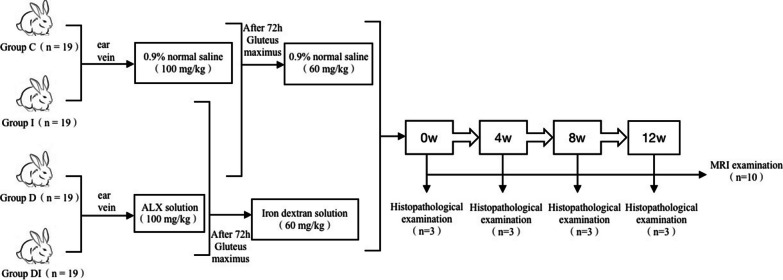
Flow chart displaying the assignment of rabbits to the study groups. Ten rabbits in each group have undergone MRI examinations immediately (week 0), and at week 4, 8, and 12 after modeling. Of the remaining nine rabbits in each group, three have been sacrificed at week 0, 4, and 8, respectively. Of the 10 rabbits undergoing MR examination, three rabbits in each group have been sacrificed following MRI at week 12. The left kidneys have been harvested for histopathological examination

72 h after intravenous injection, diabetic rabbits in the DI group and rabbits in the I group were injected with 60 mg/kg iron-dextran solution (Meryer Chemdrug, Shanghai, China) through the gluteus maximus, and rabbits in the C and D groups were intramuscularly injected with similar dose of 0.9% normal saline through the gluteus maximus (Fig. 1). Rabbits in D and DI groups were fed with a high-fat and high-sugar diet (6% lard, 2% cholesterol, and 5% sucrose) to maintain high blood glucose level, and rabbits in the C and I groups were fed with a complete diet. The rabbits were raised in separate cages for 12 weeks.

### MR examination

Magnetic resonance imaging was performed with a 3.0 T scanner (Magnetom Verio; Siemens Healthcare, Erlangen, Germany) and an eight-channel phase array body coil. The rabbits had free access to standard food and tap water until 8 h prior to the MRI examination. They were anesthetized by an intramuscular injection of 3% pentobarbital sodium (1 ml/kg) and maintained by the inhalation of isoflurane (2%-3.5% isoflurane with 100% oxygen, 1 L/min) through a breathing mask. The rabbits under anesthesia were placed on the scanning bed in the left lateral position, and sandbags were placed on both sides of the abdomen to reduce respiratory motion artifacts. Ten rabbits in each group underwent BOLD MRI of the left kidney immediately (week 0), and at week 4, 8, and 12 after modeling. Table 1 summarizes the MRI sequences and parameters. Coronal T_2_ weighted image (T_2_WI), axial T_2_WI, and axial BOLD MRI were performed. The center line of the scanning area was perpendicular to the long axis of the left kidney and was in similar section as the left renal hilus. For BOLD MRI, a set of R_2_^*^ maps were created by fitting signal intensity versus echo time to a monoexponetial function in a pixel-by-pixel scheme with motion correction between volumes: *S* = *S*_*0*_ × exp(− *TE* × R2*) [12], where *S* is the measured signal, *S*_*0*_ is the initial signal amplitude, and R2* is the effective transverse relaxation rate.

**Table 1:** Sequences and parameters of conventional MRI and BOLD MRI

Parameters	Coronal T_2_WI	Axial T_2_WI	Axial BOLD MRI
TR/TE (ms)	1400/93	1000/108	561/6.0, 11.2, 16.4, 21.6, 26.8, 32, 37.2, 42.4, 47.6, 52.8
FOV (mm^2^)	154 × 219	130 × 130	130 × 130
Matrix	126 × 256	179 × 256	218 × 256
Slice thickness/gap (mm)	3/0.9	4/0	4/0.8
Number of slices	10	8	8
Bandwidth (Hz/pixel)	781	199	331
Scan time (s)	14	98	737

*T2WI* T2-weighted imaging, *BOLD* Blood-oxygen level dependent, *TR* Repetition time, *TE* Echo time, *FOV* Field of view, *MRI* Magnetic resonance imaging

### Image analysis

**Fig. 2:**
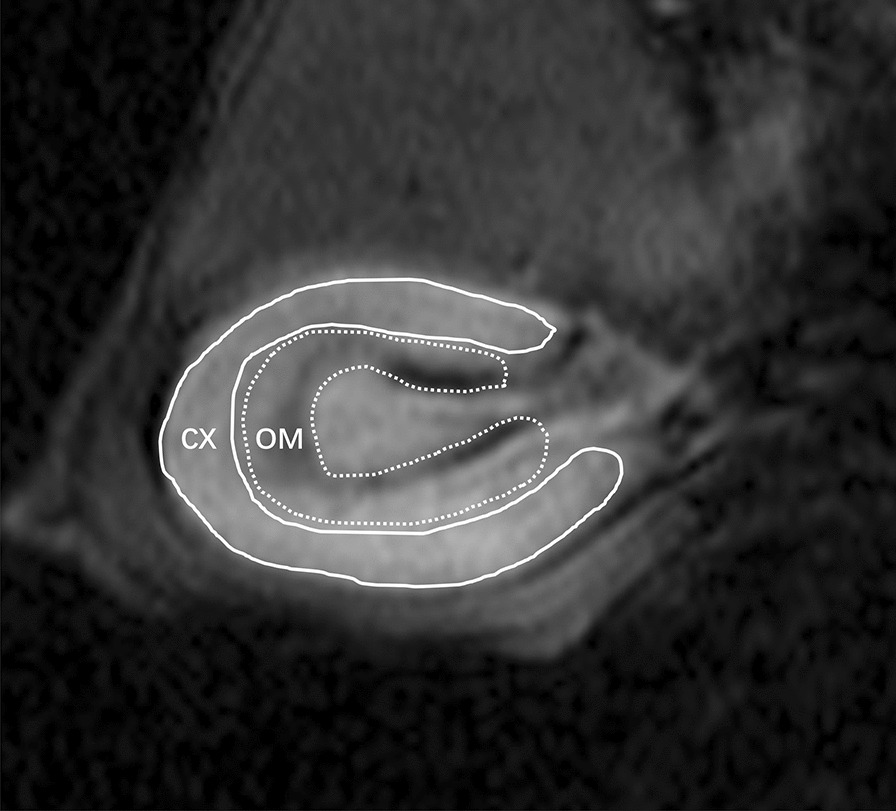
Diagram of the region of interest (ROI). ROIs were delineated along the margin of renal cortex (CX) and the outer medulla (OM) on R_2_^*^ maps

All images were analyzed on the Siemens Sygno workstation. The R_2_^*^ map on the left renal hilum section was selected for image analysis. Using T_2_W images as a reference, two regions of interest (ROIs) were delineated along the margin of renal cortex and the outer medulla on R_2_^*^ maps (Fig. 2). The R_2_^*^ values of the cortex (CR_2_^*^) and outer medulla (MR_2_^*^) were measured, and the ratios of MR_2_^*^ to CR_2_^*^ (MCR) were calculated. The inner medulla overlapped with the renal sinus fat and renal pelvis, which affected the accuracy and stability of the data. Hence, the inner medulla was not included in the ROI drawing. To test the interobserver reproducibility of the CR_2_^*^ and MR_2_^*^ values, two radiologists with 10 years and 5 years of experience (observer A and observer B, respectively) drew the ROIs independently.

### Histopathological examinations

Of the remaining nine rabbits in each group, three were sacrificed at week 0, 4, and 8, respectively. Of the 10 rabbits that underwent MRI, three in each group were sacrificed after MRI at week 12. The left kidneys were harvested for histopathological examination. The tissues were fixed in 10% formalin for 24 h and embedded in paraffin. The axial sections of the renal hilus, which corresponded to the MRI slices, were sampled vertical to the long axis of the left kidneys. The kidney slices were stained with hematoxylin and eosin (HE), Prussian blue, and Masson’s trichromatic. Kidney injury was observed in hematoxylin and eosin stained slices. The distribution of iron in renal tissue was observed in Prussian blue stained slices, which was demonstrated as a blue stained granule. Moreover, interstitial fibrosis was observed in Masson’s tingrichromatic stained slices.

### Histopathological analysis

The histopathological analysis was performed by a pathologist with more than 10 years of experience. The degree of kidney injury was assessed using a semiquantitative scoring scale (0, none; 1, area < 10%; 2, 10% ≤ area < 25%; 3, 25% ≤ area < 50%; 4, 50% ≤ area < 75%; 5, area ≥ 75%) in 10 random fields (× 200 magnification) for both renal cortex and medulla on each HE slice, and the results were expressed as the mean score in a single field.

On the Prussian blue stained slices, iron overload was quantified as the percentage of the blue stained granules over the total number of cells in 10 random regions (× 200 magnification) for both renal cortex and medulla on each slice.

On the Masson’s trichromatic stained slices, interstitial fibrosis was quantified as the percentage of the blue stained area over the total cross-sectional area in 10 random regions (× 200 magnification) for both renal cortex and medulla on each slice.

### Statistical analysis

SPSS 19.0 software (IBM, Armonk, NY, USA) was used for statistical analysis. The Shapiro–Wilk normality test was performed to determine the normality of data distribution. Quantitative data with normal distribution are presented as means ± SD. The intraclass correlation coefficient (ICC) was used to assess the interobserver reproducibility of the CR_2_^*^ and MR_2_^*^ values (0 < ICC < 0.40, indicating poor inter-observer agreement; 0.40 ≤ ICC < 0.60, indicating fair inter-observer agreement; 0.60 ≤ ICC < 0.75, indicating good inter-observer agreement; and ICC ≥ 0.75, indicating excellent observation consistency between the participants). Using the data measured by observer A, we conducted a repeated-measures analysis to compare the differences in CR_2_^*^, MR_2_^*^, and MCR values among the C, I, D, and DI groups, and the differences across different time points. The relationship between renal R_2_^*^and kidney injury, iron overload, and interstitial fibrosis for renal cortex and medulla were assessed by Pearson correlation coefficients (*r*). *P* < 0.05 was considered statistically significant.

## Results

### ***Differences in CR***_2_^*^, MR_2_^*^, and MCR values among the four groups

**Fig. 3:**
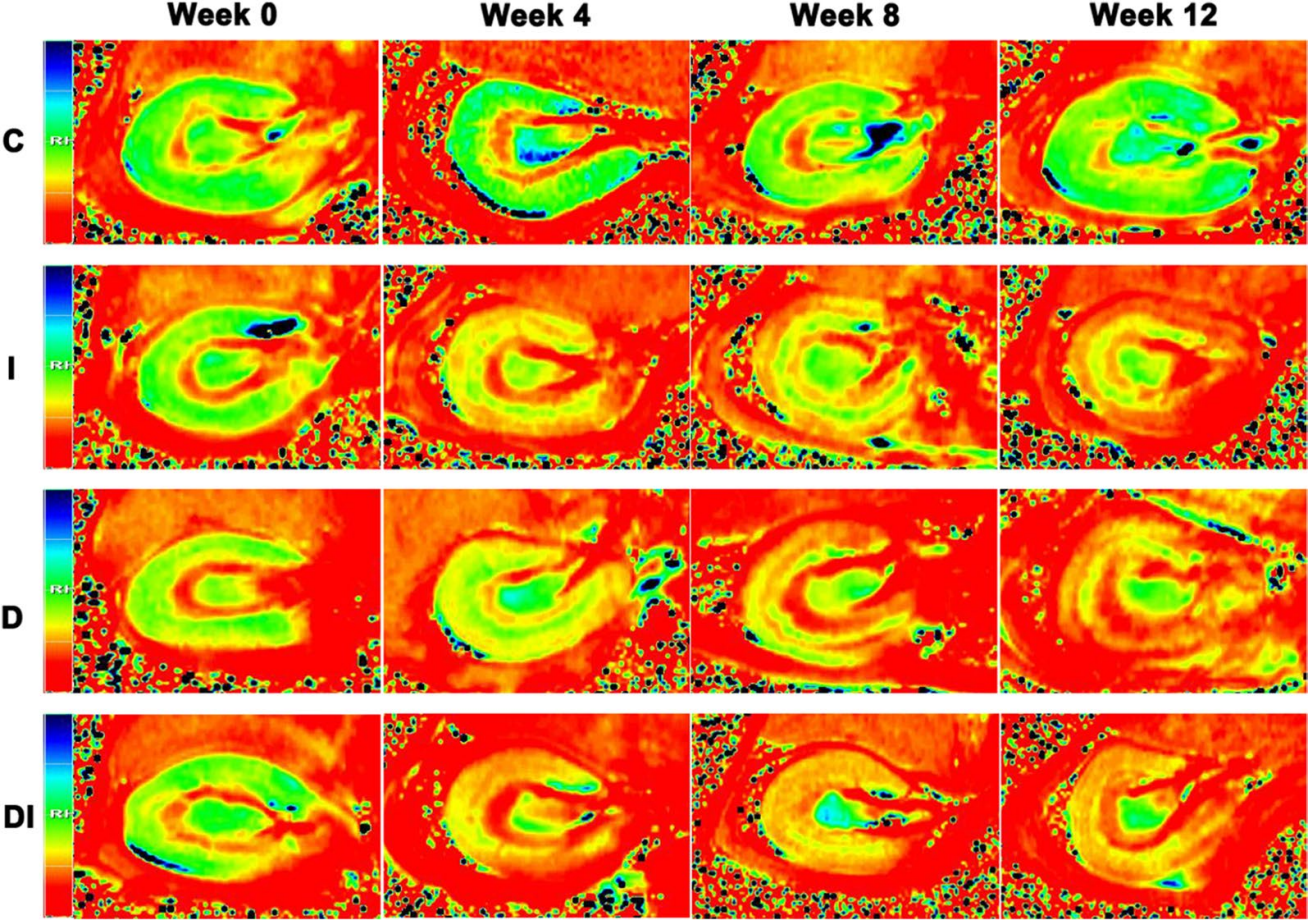
Representative R_2_^*^ maps of the left kidneys in the C, I, D, and DI groups. The boundaries of renal cortex and outer medulla are clear. While the renal cortex is green, the outer medulla is red from week 0 to 12 in the C group and at week 0 in the I, D, and DI groups. In the I group, renal cortex has changed from green to yellow-red, and outer medulla changed from red to yellow-red at week 4, 8, and 12. In the D group, the renal cortex has changed from green to yellow-red at week 4, 8, and 12. In the DI group, the renal cortex has changed from green to yellow-red, and the outer medulla has changed from red to yellow-red at week 4, 8, and 12

**Fig. 4:**
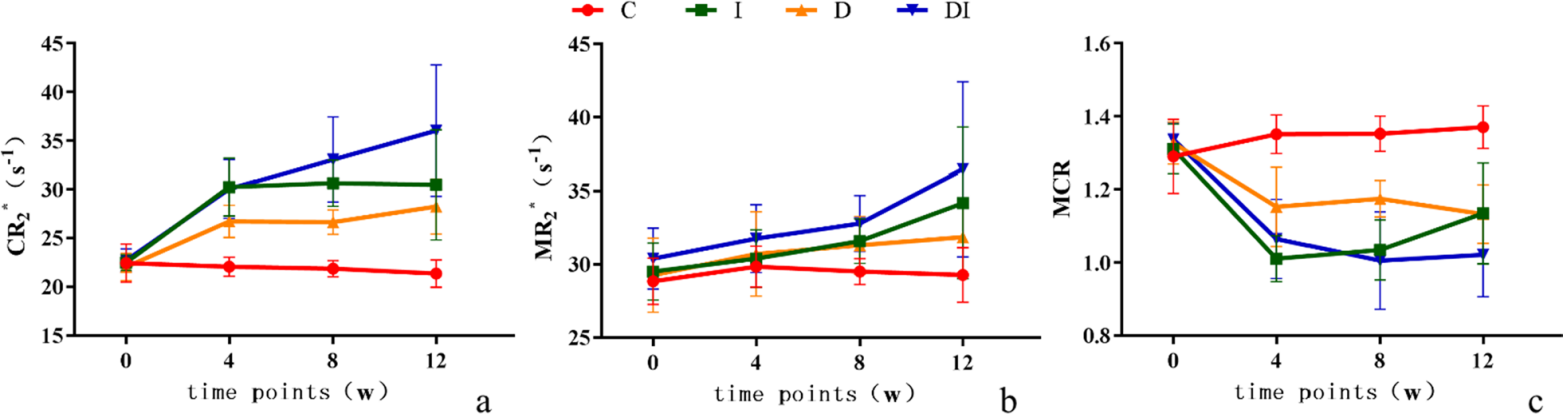
Dynamic changes of the CR_2_^*^(**a**), MR_2_^*^ (**b**) and MCR values (**c**) in the C, I, D, and DI groups

Figure 3 were representative R_2_^*^ maps for the C, I, D, and DI groups. Figures 4 and Tables 2, 3 and 4 depict the differences in CR_2_^*^, MR_2_^*^, and MCR values among the four groups at different time points. The ICC values of CR_2_^*^ and MR_2_^*^ measured by observer A and observer B were 0.915 (95% confidence interval (CI), 0.856–0.950) and 0.883 (95% CI, 0.799–0.933), respectively. At week 0, there were no significant differences in the CR_2_^*^, MR_2_^*^, and MCR values among the four groups (all *P* > 0.05).

**Table 2:** CR_2_^*^ values in the control, iron overload, diabetes, and diabetes with iron overload groups at different time points

Time	Group C (n = 10)	Group I (n = 10)	Group D (n = 10)	Group DI (n = 10)	*P* (groups)	*P* (C-I)	*P* (C-D)	*P* (C-DI)	*P* (I-D)	*P* (I-DI)	*P* (D-DI)
week 0	22.46 ± 1.94	22.51 ± 0.82	22.06 ± 1.41	22.73 ± 1.18	0.757	0.938	0.528	0.671	0.478	0.728	0.293
week 4	22.09 ± 0.97	30.26 ± 2.99	26.74 ± 1.65	30.07 ± 3.03	< 0.001^#^	< 0.001^#^	< 0.001^#^	< 0.001^#^	0.002^#^	0.855	0.003^#^
week 8	21.88 ± 0.83	30.67 ± 2.37	26.66 ± 1.24	33.10 ± 4.39	< 0.001^#^	< 0.001^#^	< 0.001^#^	< 0.001^#^	0.001^#^	0.044^#^	< 0.001^#^
week 12	21.38 ± 1.40	30.4 ± 5.65	28.25 ± 2.83	36.06 ± 6.76	< 0.001^#^	< 0.001^#^	0.002^#^	< 0.001^#^	0.292	0.012^#^	0.001^#^
*P* (time_points)	0.750	< 0.001^#^	< 0.001^#^	< 0.001^#^							
*P* (w0–w4)	0.625	< 0.001^#^	< 0.001^#^	< 0.001^#^							
*P* (w0–w8)	0.509	< 0.001^#^	< 0.001^#^	< 0.001^#^							
*P* (w0–w12)	0.486	< 0.001^#^	< 0.001^#^	< 0.001^#^							
*P* (w4–w8)	0.816	0.654	0.930	0.002^#^							
*P* (w4–w12)	0.699	0.900	0.409	0.002^#^							
*P* (w8–w12)	0.797	0.927	0.412	0.130							

Data was represented as mean R_2_^*^ value (s^−1^) ± standard deviation

*C* Control, *I* Iron overload, *D* Diabetes, *DI* Diabetes with iron overload

^#^*P* < 0.05 denotes the difference is statistically significant; *P* (groups)-values among groups C, I, D, and DI; *P* (C-I)-values between groups C and I; *P* (C-D)-values between groups C and D; *P* (C-DI)-values between groups C and DI; *P* (I-D)-values between the I and D groups; *P* (I-DI)-values between the I and DI groups; *P* (D-DI)-values between the D and DI groups; *P* (time_points) -values among week 0, 4, 8, and 12; *P* (w0–w4)-values between week 0 and 4; *P* (w0–w8)-values between week 0 and 8; *P* (w0–w12)-values between week 0 and 12; *P* (w4–w8)-values between week 4 and 8; *P* (w4–w12)*-*values between week 4 and 12; and *P* (w8–w12)-values between week 8 and 12

**Table 3:** MR_2_^*^ values in the control, iron overload, diabetes, and diabetes with iron overload groups at different time points

Time	Group C (n = 10)	Group I (n = 10)	Group D (n = 10)	Group DI (n = 10)	*P* (groups)	*P* (C-I)	*P* (C-D)	*P* (C-DI)	*P* (I-D)	*P* (I-DI)	*P* (D-DI)
week 0	28.86 ± 1.59	29.53 ± 1.95	29.28 ± 2.54	30.41 ± 2.08	0.403	0.472	0.655	0.103	0.784	0.349	0.229
week 4	29.86 ± 1.40	30.41 ± 1.97	30.73 ± 2.89	31.78 ± 2.33	0.276	0.582	0.382	0.060	0.743	0.173	0.296
week 8	29.53 ± 0.89	31.60 ± 1.53	31.33 ± 1.97	32.81 ± 1.90	0.001^#^	0.007^#^	0.019^#^	< 0.001^#^	0.704	0.107	0.049^#^
week 12	29.29 ± 1.87	34.19 ± 5.14	31.88 ± 2.50	36.47 ± 5.95	0.004^#^	0.014^#^	0.180	0.001^#^	0.229	0.236	0.020^#^
*P* (time_points)	0.738	0.006^#^	0.068	< 0.001^#^							
*P* (w0–w4)	0.270	0.332	0.110	0.131							
*P* (w0–w8)	0.450	0.024^#^	0.026^#^	0.010^#^							
*P* (w0–w12)	0.742	0.001^#^	0.054	< 0.001^#^							
*P* (w4–w8)	0.709	0.171	0.494	0.238							
*P* (w4–w12)	0.715	0.018^#^	0.455	0.004^#^							
*P* (w8–w12)	0.870	0.082	0.703	0.016^#^							

Data was represented as mean R_2_^*^ value (s^−1^) ± standard deviation

*C* Control, *I* Iron overload, *D* Diabetes, *DI* Diabetes with iron overload

^#^*P* < 0.05 denotes the difference is statistically significant; *P* (groups)-values among groups C, I, D, and DI; *P* (C-I)-values between groups C and I; *P* (C-D)-values between groups C and D; *P* (C-DI)-values between groups C and DI; *P* (I-D)-values between the I and D groups; *P* (I-DI)-values between the I and DI groups; *P* (D-DI)-values between the D and DI groups; *P* (time_points) -values among week 0, 4, 8, and 12; *P* (w0–w4)-values between week 0 and 4; *P* (w0–w8)-values between week 0 and 8; *P* (w0–w12)-values between week 0 and 12; *P* (w4–w8)-values between week 4 and 8; *P* (w4–w12)*-*values between week 4 and 12; and *P* (w8–w12)-values between week 8 and 12

**Table 4:** MCR values in the control, iron overload, diabetes, and diabetes with iron overload groups at different time points

Time	Group C (n = 10)	Group I (n = 10)	Group D (n = 10)	Group DI (n = 10)	*P* (groups)	*P* (C-I)	*P* (C-D)	*P* (C-DI)	*P* (I-D)	*P* (I-DI)	*P* (D-DI)
week 0	1.29 ± 0.10	1.31 ± 0.07	1.33 ± 0.06	1.34 ± 0.05	0.488	0.515	0.254	0.149	0.619	0.420	0.756
week 4	1.35 ± 0.05	1.01 ± 0.06	1.15 ± 0.11	1.07 ± 0.11	< 0.001^#^	< 0.001^#^	< 0.001^#^	< 0.001^#^	0.001^#^	0.172	0.029^#^
week 8	1.35 ± 0.05	1.04 ± 0.08	1.18 ± 0.05	1.01 ± 0.13	< 0.001^#^	< 0.001^#^	< 0.001^#^	< 0.001^#^	0.001^#^	0.453	< 0.001^#^
week 12	1.37 ± 0.06	1.14 ± 0.14	1.13 ± 0.08	1.02 ± 0.11	< 0.001^#^	< 0.001^#^	< 0.001^#^	< 0.001^#^	0.965	0.019^#^	0.021^#^
*P* (time_points)	0.088	< 0.001^#^	< 0.001^#^	< 0.001^#^							
*P* (w0–w4)	0.088	< 0.001^#^	< 0.001^#^	< 0.001^#^							
*P* (w0–w8)	0.078	< 0.001^#^	< 0.001^#^	< 0.001^#^							
*P* (w0–w12)	0.037^#^	< 0.001^#^	< 0.001^#^	< 0.001^#^							
*P* (w4–w8)	0.977	0.499	0.535	0.102							
*P* (w4–w12)	0.686	0.011^#^	0.670	0.362							
*P* (w8–w12)	0.694	0.034^#^	0.361	0.727							

Data was represented as mean ± standard deviation

*C* Control, *I* Iron overload, *D* Diabetes, *DI* Diabetes with iron overload

^#^*P* < 0.05 denotes the difference is statistically significant; *P* (groups)-values among groups C, I, D, and DI; *P* (C-I)-values between groups C and I; *P* (C-D)-values between groups C and D; *P* (C-DI)-values between groups C and DI; *P* (I-D)-values between the I and D groups; *P* (I-DI)-values between the I and DI groups; *P* (D-DI)-values between the D and DI groups; *P* (time_points) -values among week 0, 4, 8, and 12; *P* (w0–w4)-values between week 0 and 4; *P* (w0–w8)-values between week 0 and 8; *P* (w0–w12)-values between week 0 and 12; *P* (w4–w8)-values between week 4 and 8; *P* (w4–w12)*-*values between week 4 and 12; and *P* (w8–w12)-values between week 8 and 12

At week 4, CR_2_^*^ values in the I, D, and DI groups were significantly higher than those in the C group (*P* < 0.001). Moreover, CR_2_^*^ values in the DI group were significantly higher than those in group D (*P* = 0.003). MR_2_^*^ values among the groups had no significant differences (all *P* > 0.05). However, MCR values in the I, D, and DI groups were significantly lower than those in the C group (all *P* < 0.001). In addition, MCR values in the DI group were significantly lower than those in the D group (*P* = 0.029).

At week 8, the CR_2_^*^ and MR_2_^*^ values in the I, D, and DI groups were significantly higher than those in the C group (all *P* < 0.05). CR_2_^*^ values in the DI group were significantly higher than those in the I and D groups (*P* = 0.044, *P* < 0.001). Furthermore, MR_2_^*^ values in the DI group were significantly higher than those in the D group (*P* = 0.049). MCR values in the I, D, and DI groups were significantly lower than those in the C group (*P* = 0.007, *P* = 0.019, and *P* < 0.001). In addition, MCR values in the DI group were significantly lower than those in the D group (*P* < 0.001).

At week 12, CR_2_^*^ values in the I, D, and DI groups were significantly higher than those in the C group (*P* < 0.001, *P* = 0.002, and *P* < 0.001). CR_2_^*^ values in the DI group were significantly higher than those in the I and D groups (*P* = 0.012, *P* = 0.001). MR_2_^*^ values in the I and DI groups were significantly higher than those in the C group (*P* = 0.014, *P* = 0.001), and values in the DI group were significantly higher than those in the D group (*P* = 0.020). MCR values in the I, D, and DI groups were significantly lower than those in the C group (all *P* < 0.001). Moreover, MCR values in the DI group were significantly lower than those in the I and D groups (*P* = 0.019, *P* = 0.021).

### ***Dynamic changes in CR***_2_^*^, MR_2_^*^, and MCR values at different time points

CR_2_^*^, MR_2_^*^, and MCR values did not significantly change from week 0 to 12 in the C group (*P* > 0.05).

CR_2_^*^ values increased rapidly in the I group from week 0–4 (*P* < 0.001) and did not significantly change from week 4–12 (all *P* > 0.05). MR_2_^*^ values increased continuously from week 4–12 (*P* = 0.018) and did not significantly change from week 0–4 (*P* = 0.332). MCR values decreased rapidly from week 0–4 (*P* < 0.001) and did not change significantly from week 4–8 (*P* = 0.499). Moreover, they increased from week 8–12 (*P* = 0.034). However, the values were still lower than that at week 0 (*P* < 0.001).

In the D group, the CR_2_^*^ values increased rapidly from week 0–4 (*P* < 0.001) and did not change significantly from week 4–12 (all *P* > 0.05). MR_2_^*^ values increased continuously from week 0–8 (*P* = 0.026) and did not change significantly from week 8–12 (*P* = 0.703). In contrast, MCR values decreased rapidly from week 0–4 (*P* < 0.001) and did not change significantly from week 4–12 (all *P* > 0.05).

In the DI group, the CR_2_^*^ values increased continuously from week 0–8 (all *P* < 0.05) and did not change significantly from week 8–12 (*P* = 0.130). While the MR_2_^*^ values did not change significantly from week 0–8 (all *P* > 0.05), they increased rapidly from week 8–12 (*P* = 0.016). MCR values decreased rapidly from week 0–4 (all *P* < 0.001) and did not change significantly from week 4–12 (all *P* > 0.05).

### Histopathological findings

**Fig. 5:**
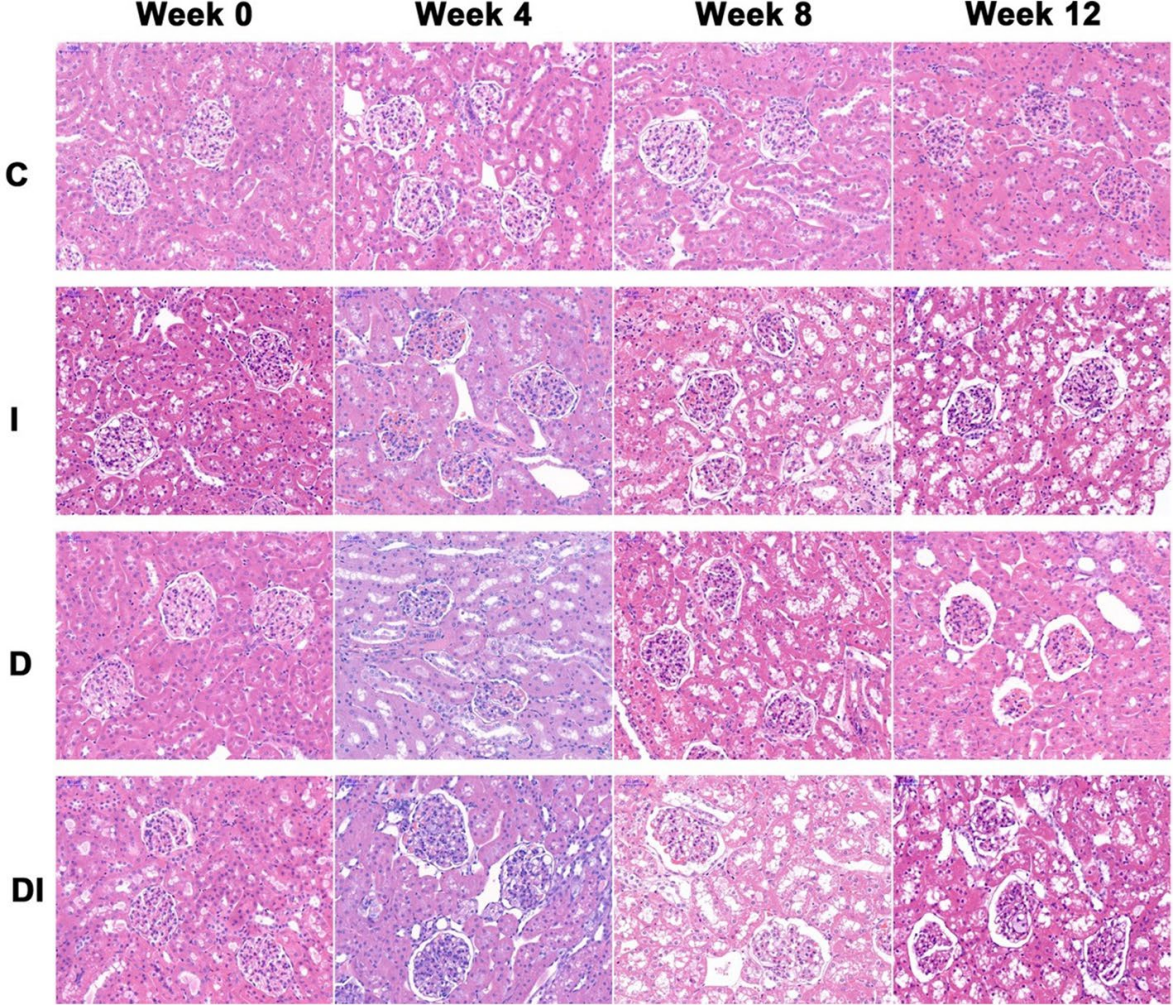
Hematoxylin and eosin staining images (× 200) of the left kidneys in the C, I, D, and DI groups at week 0, 4, 8, and 12. The morphology and structure of glomeruli and renal tubules were normal in the C group from week 0 to 12 and the I, D, and DI groups at week 0. There was edema and ballooning degeneration of few renal tubular epithelial cells in the I, D, and DI groups at week 4. The edema of tubular epithelial cells was more obvious and extensive in the I, D, and DI groups at week 8. There was necrosis of few tubular epithelial cells, the infiltration of inflammatory cells in interstitial substance, glomerular shrinkage, and interstitial fibrosis in the I, D, and DI groups at week 12. The degree of kidney injury and fibrosis was more severe in the DI group than those in the I and D groups

**Fig. 6:**
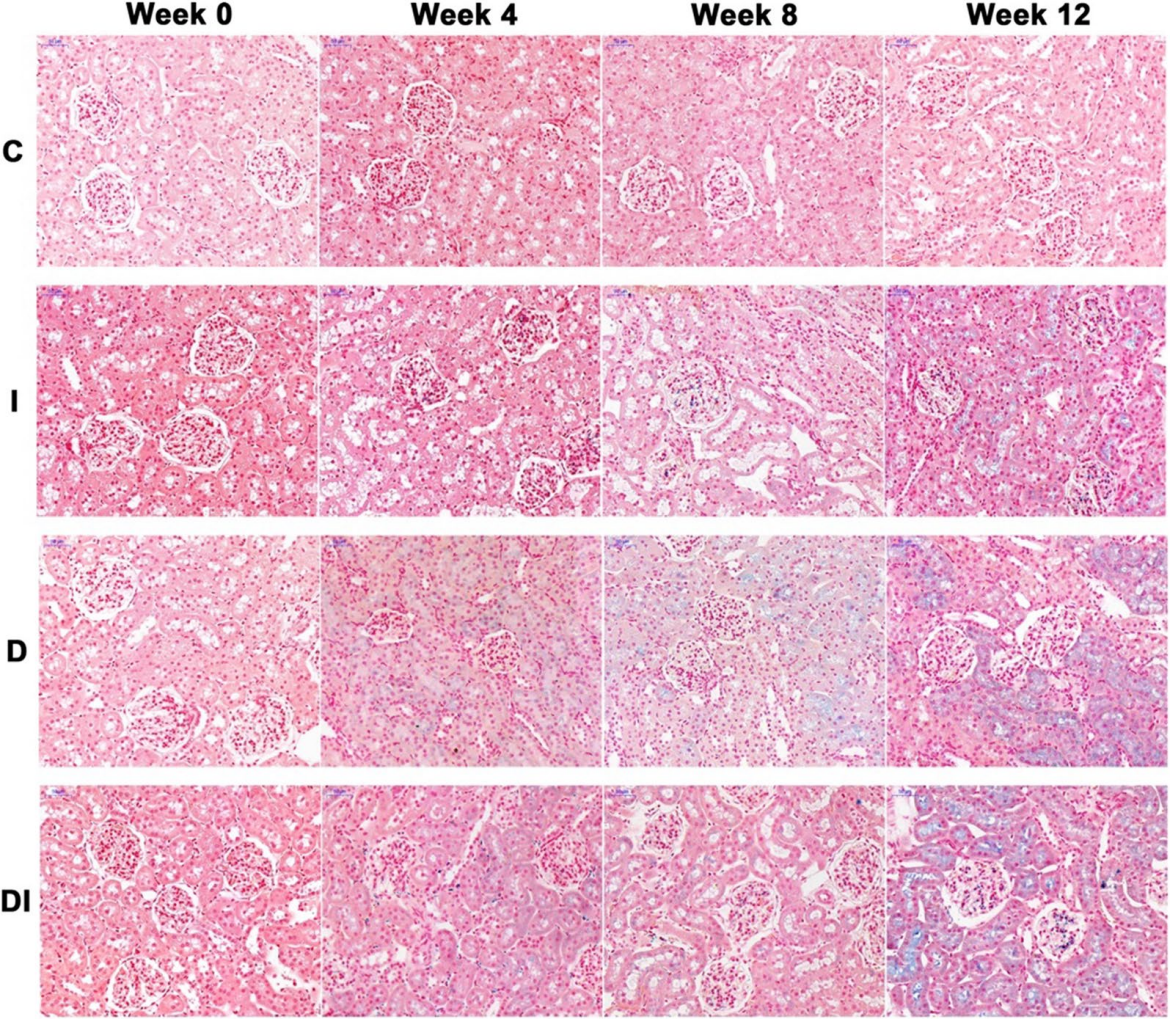
Prussian blue staining images (× 200) of the left kidneys in the C, I, D, and DI groups at week 0, 4, 8, and 12. There were no blue stained granules in the C group from week 0–12 and the I, D, and DI groups at week 0. In the I group, there were few blue stained granules in the glomeruli since week 4. A small amount of blue stained granules was observed in proximal tubule cells of renal cortex at week 12. In the D group, blue stained granules were present in the proximal tubule cells in the renal cortex since week 4. In the DI group, there were several blue stained granules in both glomeruli and proximal tubule cells since week 4. Furthermore, the amount of the granules increased significantly in both glomeruli and proximal tubule cells and was more than those in the I and D groups at week 12.

**Fig. 7:**
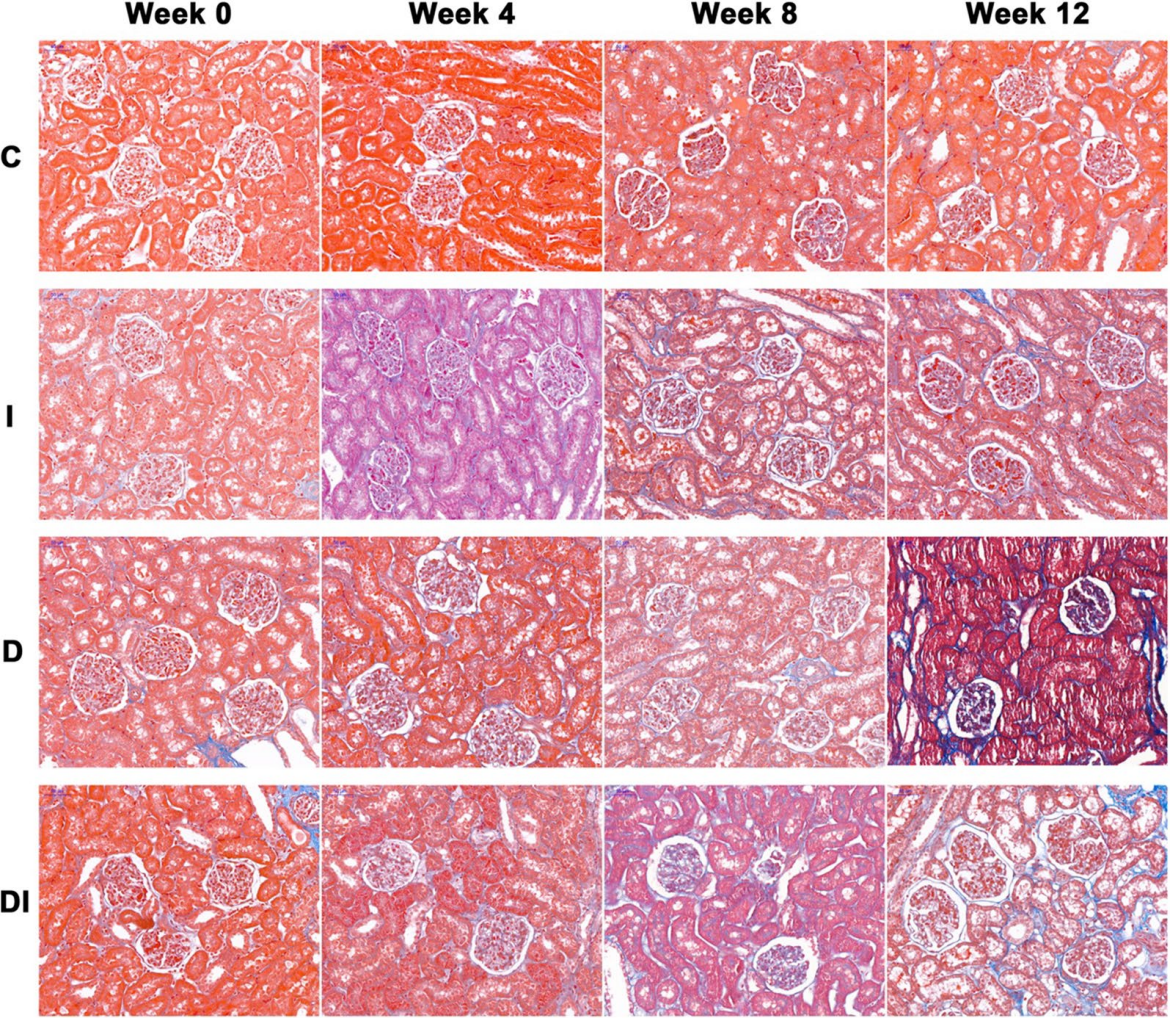
Masson’s trichromatic staining images (× 200) of the left kidneys in the C, I, D, and DI groups at week 0, 4, 8, and 12. There was no interstitial fibrosis in the C group from week 0 to 12 and the I, D, and DI groups at week 0. A small amount of blue stained interstitial fibrosis was observed in renal cortex in the I and D groups at week 8 and 12 and the DI group at week 8. At week 12, the area of blue stained interstitial fibrosis in the DI group was larger than those in the I and D groups

Figures 5, 6 and 7 depict representative histopathological images for the C, I, D, and DI groups. The morphology and structure of glomeruli and renal tubules were normal, and there was no blue stained granules and interstitial fibrosis in the C group from week 0–12 and the I, D, and DI groups at week 0.

In the I group, there were few blue stained granules in the glomeruli since week 4. Moreover, a small amount of blue stained granules was observed in proximal tubule cells of renal cortex at week 12. In the D group, blue stained granules were present in the proximal tubule cells in the renal cortex since week 4. In the DI group, there were several blue stained granules in both glomeruli and proximal tubule cells since week 4. Furthermore, the amount of the granules increased significantly in both glomeruli and proximal tubule cells and was more than those in the I and D groups at week 12. There were no blue stained granules in the outer medulla in all groups.

We observed edema and ballooning degeneration of few renal tubular epithelial cells in the I, D, and DI groups at week 4. The edema of tubular epithelial cells was more obvious and extensive in the I, D, and DI groups at week 8. In addition, we observed necrosis of few tubular epithelial cells, the infiltration of inflammatory cells in interstitial substance, glomerular shrinkage, and interstitial fibrosis in the I, D, and DI groups at week 12. The degree of kidney injury and fibrosis was more severe in the DI group than those in the I and D groups.

### Correlation between renal R2* with histopathological findings

In the I group, iron overload strongly correlated with CR2* (*r* = 0.756, *P* < 0.001), and there was moderate correlation of kidney injury with CR2* (*r* = 0.589, *P* < 0.001) and MR2* (*r* = 0.621, *P* < 0.001). A moderate correlation was observed between interstitial fibrosis and CR2* (*r* = 0.423, *P* = 0.001).

In the D group, iron overload moderately correlated with CR2* (*r* = 0.517, *P* < 0.001), and there was moderate correlation of kidney injury with CR2* (*r* = 0.539, *P* < 0.001) and MR2* (*r* = 0.605, *P* < 0.001). A weak correlation was observed between interstitial fibrosis and CR2* (*r* = 0.368, *P* = 0.024).

In the DI group, iron overload moderately correlated with CR2* (*r* = 0.670, *P* < 0.001), and there was moderate correlation of kidney injury with CR2* (*r* = 0.632, *P* < 0.001) and MR2* (*r* = 0.732, *P* < 0.001). A moderate correlation was observed between interstitial fibrosis and CR2* (*r* = 0.547, *P* < 0.001).

## Discussion

Diabetic nephropathy is a common microvascular complication of diabetes, and the most common cause of end-stage renal disease. The role of iron in the progression of diabetic nephropathy has gained more attention in recent years [4, 13, 14]. Patients with diabetes have significantly higher iron deposition and total iron content and increased urinary iron excretion than the control group. Chaudhary et al. [15] reported on upregulated expression of iron regulatory proteins in the kidneys in both type 1 and type 2 diabetic mouse, in addition to iron deposition in proximal tubular cells. A clinical longitudinal retrospective study [16] reported on an association between lower serum transferrin concentration and end-stage diabetic nephropathy in patients with type 2 diabetes. Moreover, iron deposition was observed in renal tubular epithelial cells in patients with diabetic nephropathy. In this study, CR_2_^*^ values in the D group were significantly higher from week 4–12 than those in the C group. Combined with histopathological results, iron deposition in the proximal tubule cells in renal cortex may directly trigger the increase in CR_2_^*^ values. Thevenod et al. [17] investigated the transport pathway of iron in the kidney, and demonstrated that iron was bound to transferrin. Following its filtration through the glomeruli, it got reabsorbed by the proximal tubule cells through the transferrin receptor 1. Approximately 99.3% of the iron was reabsorbed, which may be attributed to the deposition of endogenous iron predominantly in the proximal tubule cells.

Iron overload causes renal dysfunction by triggering oxidative stress and inflammatory response, which lead to a cascade of systemic and renal inflammatory processes [2]. Zhou et al. [18] discovered kidney injury in iron-overloading mice, characterized by glomerulosclerosis, tubular atrophy, and interstitial fibrosis. Moreover, they indicated that kidney injury of iron-overloading animals may be directly or indirectly mediated by excessive reactive oxygen species. Kovtunovych et al. [19] also reported similar results. A clinical study [20] confirmed that renal tubular dysfunction among patients with beta-thalassemia was closely related to iron overload. In this study, the degree of kidney injury in the I group increased gradually from week 4–12, thus indicating exogenous iron overload could directly lead to kidney injury. Interestingly, in contrast to the D group, iron was primarily deposited in glomeruli in the I group, which may be related to the following mechanisms [21]: (1) The size of exogenous iron molecules may exceed the pore size of glomerular filtration membrane (small pores 45–50 Å, large pores 75–115 Å), thereby resulting in filtration failure to the renal tubules and deposition in glomeruli; (2) In case of massive and rapid iron overload, transferrin saturates rapidly, and a part of the iron could not combine with transferrin. Moreover, it could not be filtered through the glomerulus, and eventually got deposited. In addition, we observed a small amount of iron deposition in the proximal tubules cells in the I group at week 12, which may be attributed to the imbalance of endogenous iron homeostasis secondary to kidney injury. This in turn was caused by exogenous iron overload, resulting in excessive endogenous iron deposition in the proximal tubule cells.

In this study, we observed iron deposition in the glomeruli and proximal tubule cells in the renal cortex in the DI group, and the iron deposition increased significantly with time. The findings of BOLD MRI also demonstrated that CR_2_^*^ values in the DI group were significantly higher than those in the D group. The histopathological results revealed significantly higher degree of kidney injury in the DI group than those in the D group, which in turn was related to the interaction between diabetes and iron overload. Our findings indicated that exogenous iron overload could aggravate diabetic kidney injury, consistent with the results of Gao et al. [5]. Moreover, they considered that the results may be related to oxidative and nitrification stress caused by excessive iron and reduced antioxidant potential. Chaudhary et al. [15] suggested that excessive iron may also promote the progression of diabetic nephropathy by activating the renin-angiotensin system. In addition, high CR_2_^*^ values in the DI group at week 12 may be related to interstitial fibrosis in the renal cortex. Zha et al. [9] have confirmed a positive correlation between R_2_^*^ values and renal fibrosis.

There was no evidence for iron deposition in the outer medulla in this study. However, the BOLD MRI findings demonstrated that the MR_2_^*^ values in the DI group were significantly higher than those in the D group at week 8 and 12. In other words, the increase in MR_2_^*^ values had no direct relationship with iron deposition. They might be attributed to the decrease in local oxygenation level because of the injury in the renal tubular cells in the outer medulla. Furthermore, the degree of injury of the renal tubular cells in the DI group was more severe than that in the D group. BOLD MRI has been previously conducted in animal and clinical trials to reflect the injury of renal tubules in the outer medulla, and has been associated to renal function [10, 22]. Researchers [23, 24] have reported that T_2_^*^ values could indirectly reflect the degree of kidney injury. Our findings demonstrated that CR_2_^*^ and MR_2_^*^ values displayed different trends. We calculated the MCR values to combine different dynamic trends of the cortex and outer medulla. Dynamic MCR curves in the D and DI groups were different from that in the I group. While MCR curves in both D and DI groups were presented as a ‘descending platform type’, those in the I group were presented as a ‘descending rising type’. The two forms of MCR curves may be related to different metabolic processes of endogenous and exogenous iron overload in vivo and varied mechanisms of cortical and medullary injury following iron overload.

This study had several limitations. First, the sample size was small, and we did not quantitatively analyze the histopathology results. Second, we set the ROIs manually to measure the R_2_^*^ values, and we intend to use a radiomics method in future. Third, the renal R2* is affected by physiological confounders with alterations in renal blood volume fraction being of particular relevance [25]; although we compared the differences in renal R2* at different reperfusion time points, the interactive effect of perfusion and iron deposition on renal R2* needs further research.

## Conclusion

Our study demonstrates that there is an interactive effect of diabetes and iron overload towards kidney injury. Moreover, BOLD MRI can be used to evaluate renal iron overload in diabetes and has a potential value in distinguishing endogenous from exogenous renal iron overload.

## Data Availability

The datasets used and/or analyzed during the current study are available from the corresponding author on reasonable request.

## Abbreviations

BOLD: Blood oxygen level-dependent
MRI: Magnetic resonance imaging
T_2_WI: T_2_ weighted image
ICC: Intraclass correlation coefficient
CR_2_^*^: R_2_^*^ values of cortex
MR_2_^*^: R_2_^*^ values of outer medulla
