# Correction: Evaluating renal iron overload in diabetes mellitus by blood oxygen level-dependent magnetic resonance imaging: a longitudinal experimental study

**DOI:** 10.1186/s12880-024-01243-2

**Published:** 2024-03-26

**Authors:** Weiwei Geng, Liang Pan, Liwen Shen, Yuanyuan Sha, Jun Sun, Shengnan Yu, Jianguo Qiu, Wei Xing

**Affiliations:** Department of Radiology, Third Affiliated Hospital of Soochow University, 185 Juqian Street, 213003 Changzhou, Jiangsu China

**Correction: Geng et al. BMC Medical Imaging (2022) 22:200**

10.1186/s12880-022-00939-7

Following the publication of the original article [1], the authors reported an error with regard to Figure 5. In the original article, the wrong image was used in the hematoxylin and eosin staining image of DI group at week 0, as seen below:


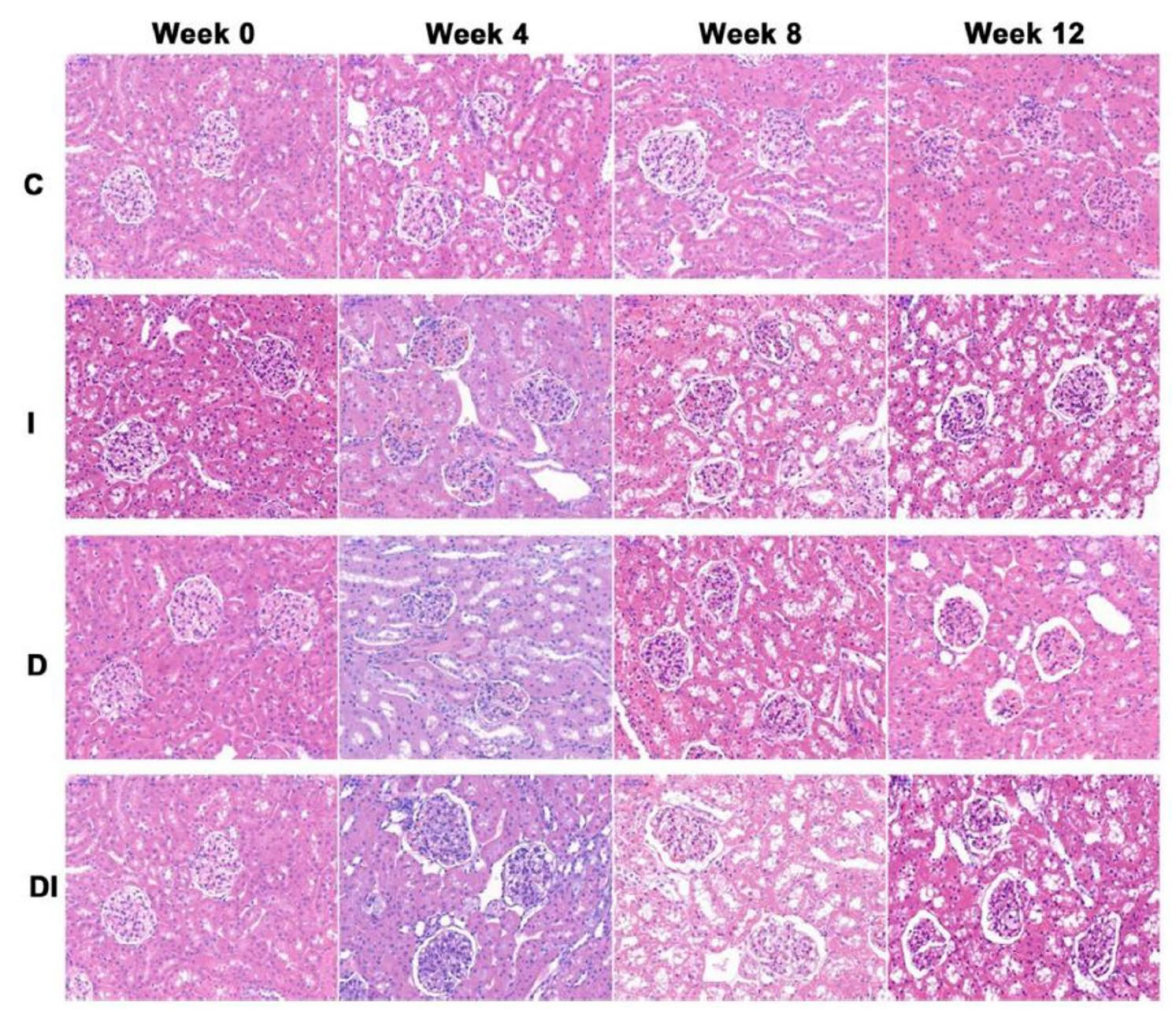


The correct Figure is as follows:

**Figure:**
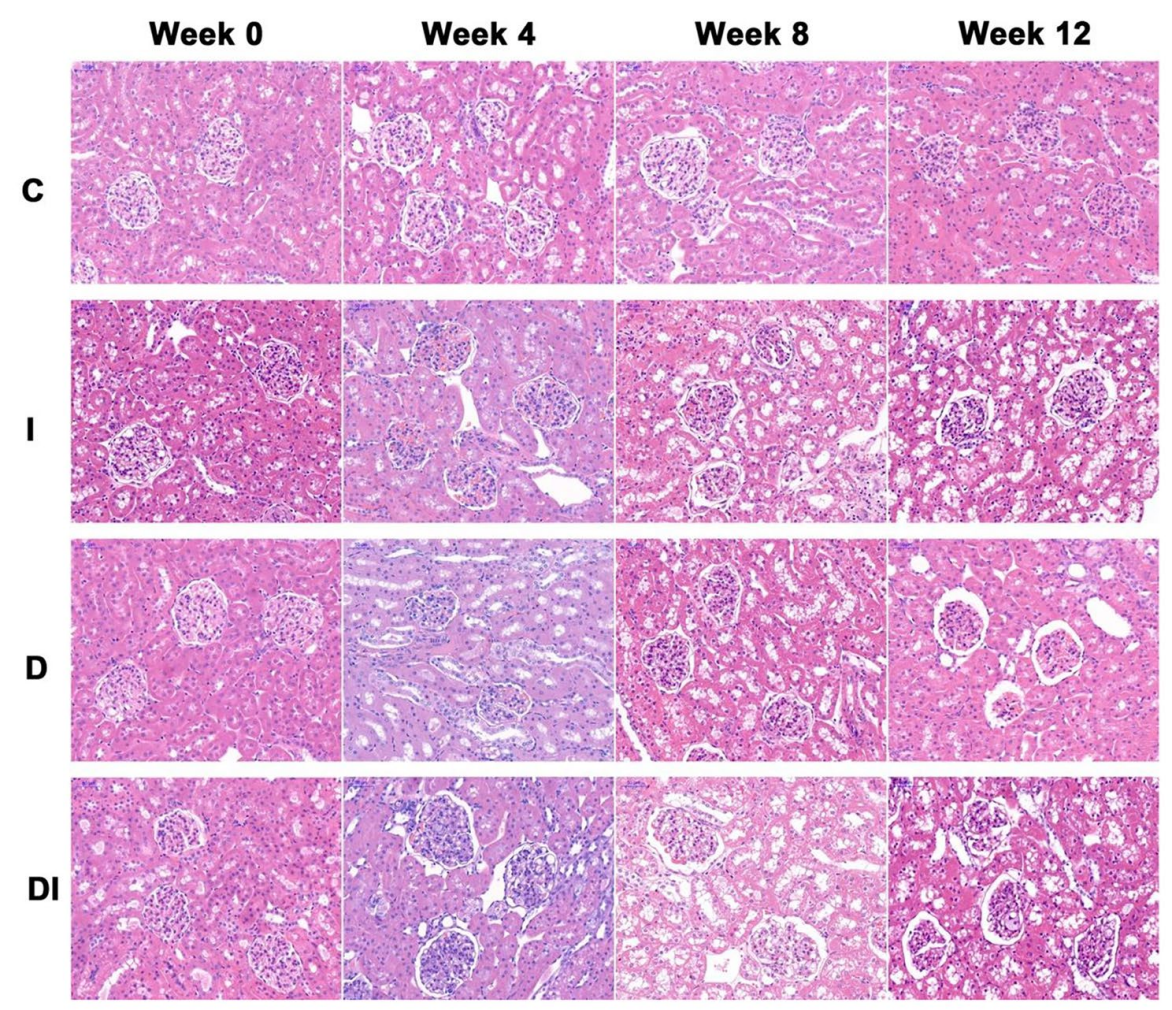

The original article [1] has been updated.
